# The Physiology of the Circulation

**Published:** 1860-02

**Authors:** John C. Dalton

**Affiliations:** Professor of Physiology and Microscopic Anatomy


NEW YORK MONTHLY REVIEW

AND

BUFFALO MEDICAL JOURNAL.

VOL. 15. FEBRUARY, 1860. NO. 9.

### ORIGINAL COMMUNICATIONS.

ART. I.—*The Physiology of the Circulation. A Course of Lectures delivered at the College of Physicians and Surgeons, New York, in the Fall Term of* 1859. By JOHN C. DALTON, JR., M. D., Prof. of Physiology and Microscopic Anatomy.

### LECTURE IV.

(SEPTEMBER 24.)

Nervous Influences affecting the Heart's Action.—Rhythmical Character of Pulsations.—Not dependent on the presence of Blood.—Experiments.—Influence on Heart of destroying Medulla Oblongata.—In Warm-Blooded Animals.—In Reptiles.—Continuance of Heart's Action after destroying Medulla and Spinal Cord.—Galvanization of Pneumogastric Nerve.—Effect on Heart's Action.—Nature of this Influence.—Effect of Narcotic Poisons on Heart.—Action of Ether and Chloroform.—Mode in which Anæsthetics prove fatal.—Action of Hydrocyanic Acid.—Of Woorara.—Varieties of Woorara.—Difference in their Mode of Action.

AFTER having thus far, gentlemen, gone through with the physical phenomena presented by the heart's action, we will to-day study some of the nervous influences which act upon the organ, and which regulate the manner in which it propels the blood through the vascular system.

The most remarkable of the nervous phenomena connected with the action of the heart, in its normal condition, is the singular regularity and persistence of its motions. The heart differs in this respect from all the other muscular organs, voluntary and involuntary. The voluntary muscles of the limbs and trunk contract only when stimulated by the nerves of motion; and the involuntary muscular organs, such as the bladder, the intestines, the œsophagus, and the uterus, are excited at certain periods by the presence of foreign bodies, or by the accumulation of their own contents. All these muscles appear to be normally in a state of quiescence; and are thrown into contraction only under the influence of an occasional stimulus. But the heart acts with a continuous and regular pulsation. Not only are its actions incessantly repeated without any apparent external stimulus, but there is a certain rhythm in the mode of their production, and its different motions follow each other in a certain definite order.

This rhythm or order of the heart's action is as follows: There are, in the time occupied by an entire cardiac pulsation, three distinct and successive periods. In the first of these, the whole heart is in a relaxed condition, receiving passively the blood which flows into it from the veins. This period occupies about two-fifths of the whole time of a pulsation. Then the second period follows, which is marked by the contraction of the auricles, and which is about one-fifth of the whole. Immediately afterward, there follows the ventricular contraction. This, as we have seen, is continuous with that of the auricles, and occupies the remaining two-fifths of the entire revolution. Then the period of rest recurs, followed by the auricular and ventricular contractions, as before.

The heart, therefore, differs from the other muscular organs in two important particulars: first, its incessantly recurring contractions and relaxations; and secondly, the regular order in which its different movements spontaneously take place.

These distinctive characters are not to be accounted for by the anatomical structure of the organ. Its muscular fibres when examined by the microscope, do not exhibit any such peculiarities as would explain their remarkable irritability, or their unusual power of continued action. The nervous filaments distributed to the walls of the heart, and the nervous ganglia with which it is provided, are similar in appearance to those met with in other internal organs. The minutest anatomical examination would not lead us to suspect that it differed from other muscles in its physiological endowments.

How are we to account for this continued and rhythmical character of the heart's action? Various explanations have been proposed, some of which are quite inadequate, others more or less satisfactory. One of them supposes that the heart is excited to contraction by the stimulus of the blood acting upon its internal surface.

This explanation is one of the most popular and plausible of all those which have been offered. According to this view, the heart is at rest so long as it remains empty, or nearly empty, of blood ; but so soon as its cavities become distended to a certain degree with the circulating fluid, the muscular fibres are stimulated to contract, just as the intestines or the œsophagus are excited by the presence of food. Immediately after a contraction, therefore, the whole heart is relaxed because its natural stimulus is absent. But as soon as the blood, flowing in from the large veins has filled the auricles to a certain point of repletion, they contract and drive the blood into the ventricles. The ventricles, thus distended, respond in their turn to the stimulus, and discharge their blood into the arteries. Once empty, both auricles and ventricles are of course relaxed, and remain quiescent until a fresh accumulation of blood again excites them to contraction in the same order as before. Thus the successive cardiac movements of systole and diastole are kept up by the alternate entrance and exit of blood in the cavities of the heart.

It is very possible that the presence of the blood in the auricles and ventricles may have a certain influence in determining the contraction of their muscular fibres. Indeed, it is very easy to see that an unnatural accumulation of blood, within certain limits, from mechanical obstruction of the circulation, gives rise to more powerful contractions of the organ, and thus appears to act as a direct stimulant to its muscular parietes. Still, this hypothesis does not wholly explain the phenomena in question. For the heart will continue to beat, even though it be empty of blood. It may even be cut out of the chest, and separated from all its connections, and of course drained completely of its fluid contents, and yet will continue to execute alternate contractions and relaxations as before. In the warm-blooded quadrupeds, these movements of the separated heart last only for a few minutes; but in the reptiles they often continue for many hours. They have been seen to go on in the eel for six hours, in the torpedo for nine hours, and in the salmon for twenty-four hours.

In order to ascertain the influence of various conditions upon the post-mortem contractions of the heart, I have tried the following experiment. Three turtles were taken, of the same species, and in the same general condition. In the first (No. 1.), the brain and medulla oblongata were broken up, and the anterior portion of the shell taken away, so as to bring into view the heart, still covered with the pericardium, and retaining its connection with the great vessels. In the second turtle (No. 2.), the brain and medulla were also broken up, and the heart, exposed as before, still retained in the pericardial sac, but the cardiac cavities were immediately drained of blood by dividing all the vessels leading to and from the heart, beyond the borders of the pericardium. In the third case (No. 3.), the heart was simply cut out of the body, drained of blood, separated from the pericardium, and left exposed upon an earthen plate.

The pulsations of the heart in these three instances continued as follows,—

| Heart's Pulsations. | No. 1. Brain and Medulla oblongata destroyed. Heart in situ. | No. 2. Brain and Medulla destroyed. Heart in situ, but drained of blood. | No. 3. Heart cut out, divested of pericardium, and drained of blood. | |
|---|---|---|---|---|
| After 20 minutes. | 2 per minute. | 12 per minute. | 6 per minut e. | |
| " 40 " | 2 " | 12 " | 8 " | |
| " 1 hour. | 4 " | 11 " | 8 " | |
| " 1 h. & 20 m. | 2 " | 11 " | 7 " | |
| " 1 h. & 40 m. | 2 " | 11 " | 10 " | |
| " 2 hours. | 2 " | 11 " | 11 " | |
| " $2\frac{1}{2}$ " | 2 " | 10 " | 12 " | |
| " 3 " | 2 " | 11 " | 10 " | Becoming feeble. |
| " $3\frac{1}{2}$ " | 3 " | 11 " | 10 " | |
| " 4 " | 4 " | 12 " | 8 " | Excessively feeble. |
| " $4\frac{1}{2}$ " | 4 " | 8 " | 0 | |
| " $6\frac{1}{2}$ " | 6 " | 14 " | | |
| " $8\frac{1}{2}$ " | 3 " | 16 " | | |

In No. 1, the movements of the heart, which had nearly or quite ceased at the 22d hour, were again excited on opening the pericardium and exposing the surface of the organ to the contact of air, when it continued to pulsate very slowly till after the 33d hour.

In No. 2, the rhythmical action was equally persistent; and forty-five hours after the commencement of the experiment, though the ventricles had stopped, the auricles still pulsated four times per minute.

It seems difficult, therefore, to attribute the movements of the heart altogether to the stimulus of the blood contained in its cavities. This stimulus, if it be the efficient cause of the cardiac contraction, must depend either on the simple *contact* of the blood with the surface of the endocardium, or on the *distention* of the cavities to a certain point by the circulating fluid. But the lining membrane of the heart is always in contact with blood, when the ventricles are partially empty and relaxed, as well as when they are full and on the point of contraction. If the simple *contact* of blood, therefore, affords the requisite stimulus, it is not easy to understand why the contraction of the ventricles should be intermittent. On the other hand, if it be the *distention* of the cardiac chambers which determines their movement, why does this movement continue after the large vessels, and even the heart itself, have been opened, and only a minute quantity of blood, to say the least, remains in its cavities?

When the heart is cut out of the chest, and so drained of blood, its movements continue, as we have seen, for a considerable time. In this case, it is true, the continued action of the organ might be referred to the unnatural stimulus of the air with which it is brought in contact. The contact of air, as is very well known, will produce contractions in the fibres of the voluntary muscles after death, and these contractions will recur at longer or shorter intervals, according to the degree of irritability possessed by the muscles. I have seen the exposed muscles of the shoulder, in a turtle, contracting occasionally for many hours after death;—and the contact of air undoubtedly has a similarly stimulating effect upon the cardiac fibres. It might be supposed, therefore, that the natural stimulus of the blood were replaced by that of the air, after excision of the heart, and that this accounted for the continued movements of the organ.

Notwithstanding this, however, such experiments are not without value, for they demonstrate that the empty and separated heart will continue to beat long enough to show, at least, that *the blood is not the only stimulus capable of maintaining its rhythmical action.* Beside, in the comparative experiment which I have just detailed, you see that in the second turtle, where the heart was drained of blood, but still contained in the pericardium, and protected from the contact of air, its pulsations continued as regular, as frequent, and for a longer time than in the heart which was separated and exposed to the air. They were also more frequent, and nearly or quite as long-continued, as in turtle No. 1, where the medullar oblongata was simply broken up and respiration brought to an end.

These facts certainly show that the contact of the blood is not the only cause upon which the *continuance* of the heart's action is dependent. It is equally impossible to explain by such an hypothesis the *rhythmical and successive character* of the cardiac movements. For the heart, when emptied of blood, still performs its pulsations in a sensibly natural manner. Its different chambers contract and relax in regular order, and in a definite succession, as before. There appears to be a certain nervous connection between its different parts, which associates their action in a particular way, independently of external or local stimulus. If the frog's heart be cut out of the body, emptied of blood, and laid upon a plate, its alternate auricular and ventricular contractions will continue for a considerable time. After these spontaneous pulsations have ceased, the heart still remaining sensitive to external stimulus, if one of the auricles be pinched between the blades of a forceps, or touched with the point of a needle, not only will the auricle itself contract, but its contraction is immediately followed by a pulsation of the ventricle, as it would be in the natural condition of the circulation. In this case there is no stimulus applied directly to the ventricle, either by the passage of blood or otherwise, and yet its movement is intimately associated with that of the auricle, and follows it in the natural order of time.

The persistent and rhythmical action of the heart, accordingly, depends upon a property inherent in the organ itself, for it survives the removal of both natural and artificial stimulus. The continued movements of the separated heart show, furthermore, that its peculiar action does not depend directly upon its connections with the nervous system. In endeavoring to explain its properties, therefore, we can only rely upon the facts which are demonstrated by experiment, and which show that the heart is distinguished from other organs by peculiar vital endowments, and that these endowments depend directly upon its own structure and upon the physiological constitution of its muscular fibres.

In every muscular organ are two distinct and opposite states or conditions, viz., a state of contraction and a state of relaxation. These two conditions are complementary to each other, and necessary to each other's existence, like the sleeping and waking conditions of the human body. For in the act of contraction, the muscular fibre uses up and expends its vital contractility or force, as a Leyden jar discharges its electricity. The contraction of the fibre, therefore, exhausts itself, and is necessarily followed by a state of relaxation. In the relaxed condition, on the other hand, the processes of nutrition go forward, which re-establish the physiological properties of the muscular fibre, and restore to it the power of contraction. In every muscle, accordingly, these two states naturally alternate with each other. During relaxation, the contractile force accumulates; during contraction, it is expended and discharged.

In all cases, furthermore, the physiological constitution and properties of the muscular tissue remain for a certain time after the death of the animal and the stoppage of the circulation. The muscular fibre, it is true, depends for its supply of nourishment upon the blood. But it still contains within itself the materials which are immediately necessary to its organization; and, until these are exhausted, it will retain its physiological properties, more or less perfectly, though the circulation may have already come to an end.

All the muscles, therefore, both voluntary and involuntary, retain their irritability for a certain time after the death of the body, and their fibres will still respond to a mechanical stimulus, by contracting, as they would do during life.

In the case of the heart, however, both during life and after death, this muscular irritability and contractility are manifested in a peculiar way. No sooner is the organ relaxed, and its nutrition momentarily re-established, than it contracts again spontaneously. Its physiological power is manifested, not by a single contraction, but by a series of successive pulsations, which follow regularly upon each other. This peculiarity of its action is to be seen after death as well as during life; in the drained and empty heart, as well as in the organ which is full of circulating blood. With the heart, therefore, it is the power of regular pulsation which becomes gradually exhausted after death, in the same way as ordinary muscular irritability disappears from the other muscular organs. When the heart is separated from the body, its pulsations, you remember, continue for a time, but afterward grow feebler and then cease. Now, if we take a heart which has thus become partially exhausted and quiescent, and irritate its substance by touching it with the point of a needle, this irritation will often give rise, not to one pulsation, but to a series of them. A single application of the stimulus, accordingly, produces a number of successive contractions, for it has the effect of exciting again the natural action of the organ, and this action is one of repeated and rhythmical pulsation.

The peculiarities of the heart's movements, therefore, can only be attributed to a power which is inherent in its own organization, and which is independent, to a certain extent, of its connection with other parts.

There are, however, certain influences exerted upon the heart by the nervous system, which we shall now proceed to study in detail. For though the heart does not derive its power of contraction from the nervous centres, since it will continue to contract when completely separated from the body, yet its movements are susceptible of being *modified* in a remarkable degree by influences communicated through the nerves. We see this often enough in the effect of moral impressions, which accelerate or retard the pulse; and Bernard has found that the heart's action may be increased by the mechanical irritation of a spinal nerve, too slight to produce any signs of pain in the animal.

The first of these influences which we shall notice, is that exerted by the destruction of certain parts of the brain and of the spinal cord, upon the heart and the rest of the circulatory system. Sudden and violent destruction of a considerable portion of the nervous centres has an immediate and depressing effect upon the heart's action. Breaking up the brain and medulla oblongata with a steel needle, in frogs and turtles, produces a partial and temporary paralysis of the heart. Afterward the organ gradually recovers, and resumes its natural action. The sudden violence inflicted on the nervous centres produces a shock, which is communicated through the nerves to the heart, and depresses its action for a time. When the heart is cut out of the body, the mutilation of the parts and the severing of the nervous connection has a similar effect, and the organ remains for a few minutes quiescent. Its pulsations then begin to return, slowly and at long intervals. As it recovers strength, its action becomes more forcible and regular, and may afterward continue, as we have seen, for many hours.

In the higher animals, injury of the medulla oblongata alone often produces a temporary excitement of the heart's action. Its pulsations become rapid and tumultuous, and can sometimes even be perceived through the walls of the chest. This, however, soon passes off, and the organ returns to its ordinary rate of movement. The entire brain has also been removed, in quadrupeds, without arresting permanently the cardiac movements. Provided, therefore, the respiration be not interfered with, we can destroy both the brain and the medulla oblongata without destroying the heart's action.

But there is a difference in the indirect effect produced upon the circulation by destruction of the medulla oblongata, in the cold-blooded and in the warm-blooded animals.

In all cases, the immediate result of such an operation is a complete stoppage of the respiratory movements. In man and the higher animals, the process of respiration is naturally exceedingly active. The movements of inspiration and expiration follow each other with rapidity, and the air in the lungs is rapidly exhausted by the blood circulating through the pulmonary capillaries. In these instances, accordingly, the destruction of the medulla oblongata, which puts a stop to respiration, soon puts a stop also to the action of the heart; for the aeration of the blood cannot be arrested without disturbing, as we have already seen, the circulation in consequence. But this influence is exerted upon the heart, not directly but indirectly. It is not, properly speaking, the destruction of the medulla oblongata that stops the heart's action. The destruction of the medulla arrests the movements of respiration, and the stoppage of respiration arrests the movements of the heart.

In the cold-blooded reptiles, however, respiration is not so continuous and unceasing a process as in the quadrupeds. The air in the lungs is exhausted more slowly, and the accumulation of carbonic acid takes place less readily and abundantly. The *mechanism* of respiration in these animals is also different. The air is not drawn into the lungs by a movement of inspiration, but is forced into them by a muscular action like that of swallowing, and is then retained there until expelled by another muscular effort. Although the respiratory movements, accordingly, be interrupted in these animals, yet the lungs still contain air enough to provide for the slow respiration which they require, and to allow for the continued circulation of the blood.

In the reptiles, therefore, while breaking up the medulla oblongata puts a stop to the movements of respiration, as it does in the human subject and in quadrupeds, it does not on that account put a stop to the action of the heart. At least, this action will go on for a considerable time, after such an injury has been inflicted.

Here is a turtle, in which the brain and medulla oblongata were destroyed three hours ago. The operation is readily accomplished in these animals, by introducing a steel needle through the foramen magnum and breaking up the nervous centres by moving the point of the instrument in various directions. The heart, which is still inclosed in the pericardium, has been exposed to view by taking away the anterior part of the shell, and removing some of the muscles in this situation. It continues to pulsate, as you observe, with regularity, though not with so much force as at first. The circulatory system is beginning to feel the influence of the exposure of the internal organs and the interruption of respiration; but it will nevertheless still perform its functions for some time longer.

There is one anatomical peculiarity in the heart of the turtle, which you may notice here. The point of the heart is attached to the inner surface of the pericardium by a loose band or bridle of fibrous tissue, which retains the organ in position, at the same time that it allows a certain amount of movement within the pericardial cavity. One or two fine red vessels can be seen running through this fibrous band, from the pericardium to the anterior surface of the ventricle. These pericardial attachments of the heart are also found in some other reptiles, such as serpents and lizards, as well as in turtles.

In this animal, gentlemen, you observe, the destruction of the brain and medulla oblongata has not arrested the action of the heart. On the contrary, the organ has continued its movements for more than three hours. I will now carry still farther the destruction of the nervous system, by breaking up the spinal cord throughout its entire length. I introduce the end of a wire into the spinal canal, and, pushing it downward, pass it through the whole length of the spinal column. The spinal cord is now completely destroyed, and yet, little or no effect is produced on the movements of the heart. These movements continue as before; or, if they show any alteration, it seems that the irritation has rather had an effect to stimulate them and increase their force. The same experiment, tried upon the frog, shows a similar result.

It is extremely interesting to notice, in this connection, that the capillary circulation is regulated in a different manner from the action of the heart, and is differently affected by injury of the nervous centres. This difference may readily be observed in the frog, by exposing the heart as usual, by cutting away the integument and muscles of the anterior portion of the body, and by placing at the same time the tongue or the transparent web of the foot under the microscope. We can then judge at any time of the condition of the capillary circulation, as well as of the cardiac movements. I have found, in this way, that if the brain and medulla oblongata alone be destroyed, the capillary circulation continues for at least two hours. But if the entire cerebro-spinal axis be destroyed, by thrusting a wire through the spinal canal, though the heart may continue to beat for three hours, the capillary circulation comes to an end within five minutes.

I will now proceed, gentlemen, to speak of a very remarkable influence exerted upon the heart through the medium of the pneumogastric nerve. This influence is of so peculiar a character that it may almost be considered as an exception to the ordinary mode of operation of the nervous system.

In experimenting upon the cerebro-spinal nerves, the most constant and uniform of all the phenomena noticed are those which follow upon the irritation or division of the nervous trunks and branches. Irritation of a nerve, by galvanism or otherwise, acts as a stimulus upon the organ to which it is distributed. Ligature or division of the nerve, on the other hand, paralyzes the organ and suspends its function. These effects are so palpable and so regular in their production, that we often rely upon the excitability of a nerve, in order to investigate the excitability of its corresponding organ. They are particularly well marked and uniform in the motor nerves, distributed to the muscles. Galvanization of a motor nerve produces contraction of the corresponding muscle. Division of the nerve, on the contrary, causes paralysis of the same part. These are the most invariable and well-understood of all the phenomena connected with the nervous system.

But a singular anomaly presents itself in the action of the pneumogastric nerve upon the movements of the heart.

Here is another turtle (a snapping-turtle), in which the brain and medulla oblongata have been destroyed, and the heart exposed, as before. The action of the heart, as you see, is still vigorous; the auricles and ventricle alternately filling and emptying themselves, with a perfectly regular and natural motion.

I now isolate the pneumogastric nerves on each side of the neck, and separate them from the neighboring parts by passing beneath them a couple of glass rods;—then, by bringing in contact with them the two poles of an ordinary galvano-electric apparatus, I pass through them a current of electricity. You see that, after the passage of the electric current has continued for a few seconds the action of the heart is arrested, and the organ remains quiescent. You will notice also the very remarkable fact that the heart stops beating, not at the moment of contraction, but at the moment of relaxation. While its movements are suspended, the organ is not in a state of spasmodic contraction, but is relaxed, and remains in that condition while the electric current is passing through the nerves.

On ceasing the galvanization, the heart remains quiescent for a second or two, and then again commences to beat;—at first with less than its usual rapidity, but in a short time it recovers itself completely, and the regular cardiac pulsations go on as before.

By again applying the poles of the battery, the same phenomena may be produced a second time. If the current be very powerful the heart stops at once. If it be only moderately strong, the pulsations continue for a short time, but recur more slowly than usual, and then cease. The ventricles dilate, but fail to contract again at their regular time, and the heart's action stops. You observe that, while in this condition, the heart is not in any degree engorged or distended with blood;—it is simply in a state of rest. Its rhythmical action ceases during the galvanization of the nerve, and re-commences after the galvanization is stopped.

Here, then, we have a phenomenon quite unexpected, and peculiar in character. Irritation of the pneumogastric nerve, instead of exciting the heart's action, stops it; and this action begins again after the irritation ceases to be applied to the nerve.

How is this peculiar influence conveyed to the heart, when the pneumogastric nerves are galvanized? Is it a reflex action, by which the irritation of the nerve is first conveyed to the medulla oblongata, and then reflected outward, by the fibres of the great sympathetic or otherwise, toward the heart? Or, is it a directly sedative action conveyed downward to the heart by the fibres of the pneumogastric nerve itself? We can settle this question in a very simple way. For if the pneumogastric nerves be divided in the middle of the neck, and the two upper extremities, which are still in connection with the brain, be then galvanized, no visible effect is produced;—but if the current be passed through the lower portions of the divided nerves, the heart stops as before, and all the phenomena take place as in our former experiment. The influence exerted by the pneumogastric upon the heart, therefore, in this instance, is direct and not reflex.

The same effect may be produced upon the heart by galvanizing the medulla oblongata, at the origin of the pneumogastric nerves. This can very readily be seen in the frog, by exposing the heart in front and the medulla oblongata behind, severing the connection of the medulla with the spinal cord, and then passing through its substance a stream of electricity, at the level where the pneumogastric nerves originate. On commencing the current, the heart stops, and remains dilated. On discontinuing the current, the cardiac pulsations begin again. But if the pneumogastric nerves be previously divided, no effect is produced on the heart by galvanizing the medulla. The influence, accordingly, whatever it may be, which arrests the heart, in this instance, is conveyed from the medulla oblongata directly to the organ itself, through the fibres of the pneumogastric nerve.

Galvanization of the pneumogastric, therefore, acts in a very exceptional way, by arresting or retarding the contractions of the heart, to which its fibres are distributed. This fact is so much the more remarkable, since if the galvanic current be applied directly to the heart itself, its effect is not to arrest but to excite the muscular contractions of the organ, as in the case of other muscles.

The *division* of the pneumogastric nerve has an equally remarkable effect upon the cardiac movements. If the two pneumogastric nerves be divided in the middle of the neck, the pulsations of the heart, instead of being suspended or retarded, are accelerated. This fact has been known for some years, and has been frequently verified by different observers. In some instances, Bernard has found the heart's movements to be doubled in rapidity after section of the pneumogastrics;—and this effect seems to be invariable in its occurrence, at least in the warm-blooded quadrupeds.

The influence of this nerve upon the heart, accordingly, if we were to judge simply from these facts, would appear to be entirely peculiar in its nature. Its direct influence would seem to be not a stimulating but a restraining one. The heart would appear to have a power of motion of its own, which is held in control and moderated by the action of the pneumogastric nerve;—so that when the nerve is excited by galvanism, its influence, being increased, overpowers completely the heart's action and holds it in check;—and when its influence is abolished, and the heart left to itself, the cardiac movements are accelerated, because the controlling power is withdrawn.

Such would be the simplest way of explaining these curious phenomena. But there are several facts which show that this explanation is not entirely correct.

In the first place, this peculiarity of action does not reside so much in the pneumogastric nerve as in the heart itself. For the pneumogastric, as we know, is distributed to other muscular organs beside the heart;—for example, to the larynx, œsophagus, and stomach. Now, it is found that the excitement or paralysis of this nerve acts upon the last-named organs in the same way as if the experiment were tried upon other motor nerves. For if the trunk of the pneumogastric nerve be galvanized, the effect of this operation is to excite the muscular fibres of the stomach, and stimulate them to contraction. On the other hand, its division paralyzes the stomach and puts a stop to its peristaltic movements. The same thing is true with regard to the muscles of the larynx and those of the œsophagus. Irritation of the pneumogastric excites these organs; its division paralyzes them. It is the heart, therefore, which has a peculiar relation to the nervous influences;—but the nerves with which it is supplied have the same general properties with those distributed to other organs.

In the second place, when the pneumogastric nerves are divided, by a transverse section, though the heart's action is accelerated it does not become more powerful. On the contrary, the *force* of the cardiac pulsations is diminished, at the same time that they become more rapid. Indeed, it is a general rule with regard to the movements of the heart, as we shall see hereafter, that whenever their rapidity is increased they become less powerful;—and this rule holds good in the present instance. Furthermore, it may be often noticed that galvanization of the pneumogastric nerves does not appear to diminish the contractile power of the heart, though it suspends its manifestation. For after the galvanization is stopped and the heart's action begins again, the contractions of the organ are quite as powerful as ever, and even seem to have acquired a greater force during the period of repose.

These phenomena, therefore, cannot be entirely explained by attributing a directly sedative influence to the pneumogastric nerve.

Various other explanations have been offered by different observers, in order to account for the singular phenomena which we have just witnessed. It has been thought by some that the galvanization of the pneumogastric nerves acts by exciting contraction in the blood-vessels ramifying in the tissue of the organ. These vessels, accordingly, being spasmodically contracted and emptied of their contents, the natural stimulus of the blood is withdrawn from the muscular fibres of the organ, and they therefore cease to contract. On the other hand, when the pneumogastrics are divided or tied, the capillary vessels and small arteries of the heart are supposed to be paralyzed and dilated. A larger quantity of blood than usual is thus allowed to accumulate in the tissue of the heart, and its muscular fibres consequently contract oftener than before.

I do not know that there are any direct proofs in favor of this explanation. If it were correct, the heart would necessarily be in an anæmic or exsanguine condition during the suspension of its movements by galvanization;—but its appearance does not usually indicate this to be the case.

Milne Edwards has proposed another and quite different theory from that just mentioned. He believes that there is in the living body a certain vital activity or force pervading the different organs, and manifesting itself in the vital phenomena. He believes, also, that there is a kind of balance or unstable equilibrium between the quantities of this force residing in different parts;—so that if it be suddenly increased in any one organ, the superabundance of force, so generated, will flow outward toward the neighboring parts;—and, on the other hand, if it be suddenly exhausted or expended in any part, a compensating flow of nervous force will take place toward that point, causing a diminution of vitality in the adjacent organ, or those in immediate nervous connection with it. When a stream of galvano-electricity, accordingly, is passed through the medulla oblongata or the pneumogastric nerve, it is supposed to exhaust momentarily the nervous vitality at the point indicated;—and a corresponding drain is made upon the heart, to compensate for this loss, and the cardiac movements are therefore diminished in rapidity. If the galvanization of the medulla or of the nerve be excessive, so great a diminution takes place in the vitality of the heart, that its pulsations are arrested altogether.

This theory, proposed by Milne Edwards, is an ingenious one, and would be quite satisfactory if there were no other facts to be considered. But unfortunately it fails altogether in explaining the principal anomaly of the phenomena in question. For what we find most difficult to understand is this:—*Why does galvanization of the pneumogastric act upon the heart in a different way from that in which galvanization of the same or other nerves acts upon other muscles?* Galvanization of a nerve, in general, does not exhaust the nervous force, as a primary effect, but excites it. If we subject the sciatic or the facial nerve to even a very powerful current of electricity, the effect is to produce instant contraction in all the muscles connected with it; and exhaustion only follows as a secondary consequence, after the nerve has been repeatedly excited by continued and violent stimulus. Beside, the pneumogastric nerve itself, when galvanized, excites contraction, as I have already mentioned, in the other muscular organs to which it is distributed, and produces relaxation only in the heart.

The various theories, therefore, which have been proposed in connection with this subject, are none of them quite satisfactory; and we shall perhaps be obliged to rest contented with knowing the bare fact that the heart acts in this peculiar manner, and is influenced as we have seen by the galvanization of its nerves.

If I were to offer, however, an explanation of my own to account for these peculiarities, it would be as follows. I believe, as already intimated, that the phenomena in question do not depend upon any peculiar action of the pneumogastric nerve, but upon a peculiarity in the physiological organization of the heart itself. The pneumogastric does not act differently from other nerves, but the heart acts differently from other muscular organs.

In the first place, division of the pneumogastric nerve in reality diminishes the action of the heart. It is true that the cardiac pulsations are more frequent after section of the nerve; but it is also true that they are less powerful. This fact has been noticed repeatedly by experimenters, and is at least equally important with the other. We have here, therefore, two results following upon section of the pneumogastrics. First, diminution in the force of the heart's pulsations; second, increase in their frequency. These two phenomena, in fact, almost invariably accompany each other, whatever may be the exciting cause upon which they depend. In most pathological conditions, as well as in physiological experiments, we find that an enfeebled condition of the heart is accompanied by an unusually rapid pulsation. The increased rapidity of pulsation, therefore, after division of the pneumogastrics, is simply in consequence of a diminition in the power of the heart; and is to be regarded as a secondary symptom, not as an immediate effect of the operation.

On the other hand galvanization of the pneumogastrics diminishes the frequency of the heart's pulsations, and if sufficiently intense suspends them. But at the same time, the cardiac contractions, though slower than usual, are more powerful while under the influence of the galvanization. This increased force of the heart's action may sometimes be appreciated by the eye when the organ is exposed to view; and after its pulsations have been suspended for a time by a powerful galvanic current, they may be seen to recommence, as I have already mentioned, with greater force and vigor than before. Now, the heart, as we know, has the power of contracting at regular intervals by virtue of its own force or physiological constitution. And this force, which is generated in the heart by its own nutrition, is discharged or expended at each effort of contraction. The contractile power of the organ, therefore, which is discharged at each pulsation, accumulates in the intervals of contraction. I believe that galvanization of the pneumogastric nerve increases directly the contractile power of the heart; and that it diminishes the frequency of the pulsations, because it stimulates the nutritive process which takes place in their intervals.

In point of fact, when the heart is vigorous and powerful it always pulsates slowly; and when enfeebled by injury or disease it pulsates more rapidly. However we may explain it, it is certainly true that when the entire system is in its most healthy and efficient condition, the heart's pulsations are comparatively slow, steady, and powerful; and an increase in force is naturally accompanied by a diminution in the rapidity of its action. We may suppose, therefore, that when galvanization of the pneumogastric nerve diminishes the frequency of the heart's pulsations, and at the same time increases their force, it only acts in the same way with any other cause which might stimulate the nutrition of its tissue.

But we will now leave these theoretical discussions, and pass to the consideration of certain poisons, and the influence which they exert upon the heart's action.

The tendency of most of the *narcotic* poisons is to stop respiration without affecting directly the movements of the heart. We know that opium, more particularly, has this effect. If a patient be suffering from an overdose of opium, or any of its preparations, one of the most striking symptoms is retardation of the respiratory movements. If the dose be excessive, the respirations will gradually fall from eighteen to ten, six, five, and even four per minute. If the narcotism be carried beyond this point no hope of recovery exists, except in the employment of artificial respiration. The heart, during thsi time, suffers but little. Its pulsations continue, and will continue if the respiratory movements do not fail from the action of the narcotic. If artificial respiration, accordingly, be kept up for eight, ten, or twenty-four hours, as the case may be, the effect of the poison during this period gradually wears away. The natural movements of respiration then recommence, and the patient recovers.

But there are some poisons which seem to act more immediately and directly upon the heart itself; and among these I believe to be the anæsthetic agents, ether and chloroform.

It is very true that both these substances will sometimes produce a stoppage of respiration without arresting the motion of the heart. But in these instances the stoppage of respiration is merely temporary; and is simply due to the profound state of anæsthesia which has been produced. In a few seconds the excessive narcotic influence is abated, and the animal begins breathing again, without having suffered any permanent injury. It is very curious to see the respiratory movements recommence spontaneously after they have been stopped by ether or chloroform, the pulsations of the heart continuing all the while without interruption.

These are the ordinary effects of the anæsthetic agents. But beside this, they are liable also to exert a specific poisonous action, by paralyzing the heart. Now, we know that if respiration be stopped, and the heart continue to pulsate, recovery will take place. But if the heart stops, it will do no good to keep up the respiration, and the animal necessarily dies. In all cases where death follows from the influence of the anæsthetics, experiment shows that the fatal effect is due to a direct and sudden paralysis of the heart.

This effect is infinitely more apt to take place with chloroform than with ether; but it may be produced with either of these anæsthetics, at least in operating upon small animals.

In order to show the influence of ether in producing paralysis of the heart, I will etherize these two cats. In one of them the etherization will be simply carried to the point of insensibility; in the other it will be so given as to produce, if possible, a fatal effect.

I now etherize the first animal. He struggles a little at first, but soon becomes more quiet, and is now under the influence of the anæsthetic. The movements of respiration, you observe, are exceedingly quiet and regular. The heart's pulsations are rapid, but otherwise quite natural and vigorous.

I will now break up the medulla oblongata in this animal, by introducing a steel instrument through the foramen magnum. You see the respiratory movements instantly cease. I now proceed to open the chest rapidly and expose the heart to view. The organ, you observe, is still pulsating, and pulsating with a great deal of vigor. These pulsations will undoubtedly continue for several minutes, notwithstanding the double influence of the administration of the anæsthetic, and the violent stoppage of respiration. You can even repeat here, the observations which we made at the last lecture, on the swelling of the heart and the distention of its cavities after suspension of respiration. You see that the accumulation of blood is beginning to take place, and that the pulsations are becoming labored in character. It is now a minute and a half since the medulla was destroyed, and fully a minute since the chest was opened; and yet the cardiac movements continue with a great deal of regularity, and will evidently continue, in some sort, for a considerable time longer. It is only at the end of three minutes that they begin to be irregular, and show the approaching condition of over-distention and paralysis.

It is evident, therefore, that in this instance the ether did not exert any directly poisonous effect on the heart.

Now, in this second animal, I endeavor to administer the ether so as to produce death. By a prolonged and excessive anæsthesia, we can at almost any time produce a fatal result, in the smaller animals; but this accident often happens, especially in the employment of chloroform, without our expecting it, and from some cause which we cannot readily explain. In this instance, you will observe, I take pains not to prevent the free access of atmospheric air; I wish merely to produce a thorough and complete action of the anæsthetic vapor, unmixed with any influence of obstructed or deficient respiration.

On feeling the walls of the chest, I find that the heart still continues to pulsate with its usual regularity. The respiration, which was suspended for a moment, immediately recommences. But, on continuing the etherization, the respiratory movements become less forcible than before. I feel the chest still expanding and collapsing regularly, but more feebly. The heart's pulsations, also, are less powerful, though rapid. You observe, at this time, some spasmodic movements of the voluntary muscles, which however are soon over. The heart has stopped;—or at least, its pulsations can no longer be felt through the walls of the chest,

I will now open the chest as rapidly as possible, and expose the thoracic organs. You observe the contractions of the heart continue, but they are infinitely less powerful than in the other instance. In fact, notwithstanding the difference in time which has elapsed since the chest was opened, in the two animals, you see there is not much, if any, difference in the action of the heart. In both, the cardiac pulsations are now excessively feeble; but in the first cat, they are still nearly or quite equal, in force and regularity, to those in the second.

In the former instance, the heart stopped simply on account of the suspension of respiration; in the second case, it was directly paralyzed by the influence of the ether.

There are two other poisons, which have a very different effect upon the heart, to which I will finally direct your attention. These are hydrocyanic acid and woorara. Hydrocyanic acid acts in very much the same manner as ether and chloroform, only more promptly and certainly; that is, it suspends directly the action of the heart. This is to be seen very distinctly in the frog, and other reptiles, in which the action of the heart is naturally very persistent. Here, for example, is a frog, in which I break up the medulla oblongata, and then cut away the parietes of the chest, so as to expose the heart. You see that the organ continues to pulsate with almost its accustomed regularity; its action not being so closely dependent on respiration, as in the warm-blooded animals.

Here, however, is a frog which has been poisoned with hydrocyanic acid. On cutting away the walls of the chest, you see that the heart is perfectly motionless. It has been paralyzed by the action of the poison.

Woorara, on the other hand, has a very different action. If a dog be poisoned by injecting a small quantity of this substance in solution into the blood-vessels, or into the areolar tissue, the first effect of its operation is to produce a partial paralysis of the voluntary muscles. The animal is suddenly affected with a trembling of the limbs, accompanied with stumbling and irregularity in the gait. He soon after falls powerless upon the floor, and the paralysis of the voluntary muscles becomes complete. In a short time it begins also to affect the muscles of respiration, and the respiratory movements become more and more imperfect, and at last cease altogether. During this time there is no sign of pain, and no convulsions, but there is simple paralysis of the motor nerves, and a consequent want of action in the muscles. Woorara, however, exerts no direct action upon the heart. The cardiac pulsations not only continue, but remain quite as powerful after poisoning with woorara as before. In the warm-blooded animals, accordingly, we can maintain the action of the heart by artificial respiration, and thus study its movements for an indefinite length of time.

Here, gentlemen, you see a frog, which has been poisoned with woorara for the last two hours. The animal is, to all appearance, completely dead. There is no sign of consciousness, no voluntary movement, no reflex action. But on opening the chest, you see the heart is still performing its regular alternate movements of contraction and relaxation.

You will notice, also, in these two frogs, another peculiarity which distinguishes the action of the two poisons. In the frog killed by hydrocyanic acid, though the heart has stopped, the blood is everywhere of a scarlet color; and the heart, as well as the tissues generally, has a bright rosy color. This seems to depend upon a specific effect of hydrocyanic acid upon the blood. In the frog poisoned with woorara, on the other hand, the blood is everywhere of a dark venous hue, from want of respiration; and the heart and all the tissues present a dusky bluish or leaden tinge.

There are several varieties, however, of this curious poison which are so different in their mode of operation, that I will ask your attention to some of the most remarkable. Here is a specimen which came from South America only a year or two ago. It acts by stopping the voluntary movements, destroying, at the same time, consciousness, volition, sense, and particularly the movements of respiration,—everything, in fact, but the action of the heart. Most kinds of woorara which have been used for experiment act precisely in this way. I have here a specimen which was given me by Dr. John W. Greene. It was brought from South America, nearly twenty years ago, and still retains its original vigor. It destroys all volition and consciousness, but leaves the action of the heart undisturbed.

Here is another specimen, which was brought from Venezuela in 1858, and which I obtained through the politeness of Dr. E. Acosta. This has also the effect of stopping all voluntary motion, but, so far as I can judge, does not interfere, to the same extent, with sensation and consciousness;—

so that although the animal cannot struggle, he still seems capable of feeling pain to a considerable degree. These differences in the action of the poison may account for the various results which different experimenters have met with, in using it. It is not at all unlikely, indeed, that there may be as many different kinds of woorara as there are tribes of Indians who manufacture it.

Here is a gourd, filled with the poison, as it is prepared by the natives. It is sometimes put up in this way, sometimes in little stone jars, sometimes smeared on the outside of pieces of reeds.

I have here, however, another variety of woorara, which is exceedingly remarkable, and differs from all the others in its mode of operation. It is a kind called by the natives "Corroval." I am indebted for this specimen to the kindness of Dr. Wm. A. Hammond, of the United States Army, who received it from South America. It is said to have been obtained originally from the River Darien, in New Granada.

The Corroval seems to act in a precisely opposite manner to the other varieties of woorara. The rest, as we have seen, paralyze the voluntary muscles, but leave the heart unaffected. This, on the contrary, stops the action of the heart, and has little or no direct influence on the voluntary motions. Dr. Hammond has found that in frogs the heart stops beating in from five to seven and a half minutes after the inoculation of the poison; while the voluntary motions continue for twenty to twenty-five minutes, and reflex movements for over three quarters of an hour. He considers, therefore, the cessation of the voluntary and reflex movements as a secondary consequence only, and considers that the direct action of the poison is exerted upon the heart itself.

Here are two frogs, which were poisoned, a few moments before the lecture commenced, by this variety of woorara. You see that in both these animals the pulsations of the heart have entirely ceased. I will now inoculate another frog with the same kind of poison, and we will then see the rapidity with which this effect is produced. I will first break up the brain and medulla oblongata, so as to destroy all consciousness and voluntary motion; and then, after opening the chest, you see the heart still beating regularly, in its normal position. An incision is then made in the back, and a small quantity of corroval, in solution, injected beneath the integument. At the same time, in order to observe the comparative effects of this poison, I will break up the brain and medulla in another frog, and after cutting open the chest, allow both animals to remain with the heart exposed.

At the end of a minute and a half, you already see a sensible diminution of the cardiac movements, in the poisoned frog. The pulsations grow less and less active, as the poison continues to be absorbed. They have now entirely ceased, while in the other animal the heart's action continues as regular and vigorous as before.

Such is the action of some of the most important varieties of poison upon the action of the heart. At the next lecture we shall pass on to the study of the arteries and the arterial circulation.

